# Analytical cryptanalysis upon *N* = *p*^2^*q* utilizing Jochemsz-May strategy

**DOI:** 10.1371/journal.pone.0248888

**Published:** 2021-03-24

**Authors:** Nurul Nur Hanisah Adenan, Muhammad Rezal Kamel Ariffin, Faridah Yunos, Siti Hasana Sapar, Muhammad Asyraf Asbullah

**Affiliations:** 1 Institute for Mathematical Research, Universiti Putra Malaysia, Serdang, Selangor, Malaysia; 2 Department of Mathematics, Faculty of Science, Universiti Putra Malaysia, Serdang, Selangor, Malaysia; University College of Engineering Tindivanam, INDIA

## Abstract

This paper presents a cryptanalytic approach on the variants of the RSA which utilizes the modulus *N* = *p*^2^*q* where *p* and *q* are balanced large primes. Suppose $e\in Z^{+}$ satisfying gcd(*e*, *ϕ*(*N*)) = 1 where *ϕ*(*N*) = *p*(*p* − 1)(*q* − 1) and *d* < *N*^*δ*^ be its multiplicative inverse. From *ed* − *kϕ*(*N*) = 1, by utilizing the extended strategy of Jochemsz and May, our attack works when the primes share a known amount of Least Significant Bits(LSBs). This is achievable since we obtain the small roots of our specially constructed integer polynomial which leads to the factorization of *N*. More specifically we show that *N* can be factored when the bound $\delta <\frac{11}{9}-\frac{2}{9}\sqrt{4+18\gamma }$. Our attack enhances the bound of some former attacks upon *N* = *p*^2^*q*.

## 1 Introduction

Secure communication up till the 70’s was executed through symmetrical ways. In other word, both of the encryption and decryption processes used the same key. Later in 1978, the first assymetric cryptosystem went public and solved the problematic issue of distributing keys. This cryptosystem used different keys to encrypt and decrypt the data. It is known as the RSA cryptosystem [1]. The construction of the RSA algorithms comprise of key generation, encryption and decryption. During the key generation process, two large balanced primes *p* and *q* are generated and the modulus *N* = *pq* is computed. Next, let *e* be a random integer such that gcd(*e*, *ϕ*(*N*)) = 1 where *ϕ*(*N*) = (*p* − 1)(*q* − 1) is the Euler totient function. Let *d* be its multiplicative inverse of *e* such that *ed* ≡ 1 mod *ϕ*(*N*). Let (*N*, *e*) be publicised for encryption purpose while *p*, *q*, *ϕ*(*N*), *d* are kept private. For decryption process, private parameter *d* is needed. The mathematical difficulty of the RSA cryptosystem relies on the hardness of solving the integer factorization problem on *N* = *pq*, solving the key equation *ed* − *kϕ*(*N*) = 1 and solving the RSA diophantine key equation that is, *C* ≡ *M*^*e*^ mod *N*. Up until today, the RSA cryptosystem has remained secure.

In 1990, [2] found out a potential weakness on this cryptosystem. He proved that if $d<\frac{1}{3}N^{\frac{1}{4}}$, then one can factor *N* by using the continued fractions expansion method. In the following years, more resarchers worked on the same objective as [2] and managed to enhance the bound *d*. Later in 1996, [3] came out with an astounding method that is very useful to find the roots of either univariate or multivariate polynomial. Since then, this method has been used extensively in both cryptography and cryptanalysis. [4] utilized this method in their attack and they improved the bound of [2] up to *d* < *N*^0.292^.

Another potential weakness upon the RSA cryptosystem is when there is leaked information regarding either the MSB(s) or LSB(s) of the private keys which is known as partial key exposure attack. In 1998, [5] proved that the whole value of *d* could be retrieved if a quater of *d* is known [6], and [7] also showed that if the primes share either MSB(s) or LSB(s), then the modulus can be factored in polynomial time. Later in 2014, [8] published an attack on RSA cryptosystem when the primes share the LSB(s) and there exists two public exponents such that their private exponents share their MSB(s).

Multi-Power RSA is one of the variants of the RSA whereby the modulus *N* = *p*^*r*^ *q* for *r* ≥ 2 is utilized. This type of modulus provides advantage for both key generation and the decryption algorithms provided the Chinese Remainder Theorem is utilized [9]. Among cryptosystems that utilize this fact are designs by [10–12]. Through their papers, the designers managed to show that their cryptosystems had low computing costs compared to the standard RSA.

As such, the study of the Integer Factorization Problem of *N* = *p*^*r*^ *q* becomes important. [13] proved that *N* = *p*^*r*^ *q* is factorable for large *r*, when *r* ≅ log *p*. Since then, many attackers made an attempt to cryptanalyse the multi-power RSA modulus. For instance, [14] showed that the modulus *N* = *p*^*r*^ *q* is more vulnerable compared to *N* = *pq*. For *r* = 2, the author proved that *N* can be factored if *d* < *N*^0.292^. In 2014, [15] presented his proof that *N* = *p*^2^*q* can be factored by using lattice reduction techniques provided *d* < *N*^0.395^.

### 1.1 Our contribution

We are working on the same purpose as the previous researchers which is to find other weakness of the RSA in order to enhance its security. Therefore in this paper, we present an attack on the modulus *N* = *p*^2^*q* where the primes share a known amount of LSB(s). Note that this is an extended result from [8]. We apply the strategy of Jochemsz and May to find small roots of our integer polynomial and show that the modulus *N* can be factored when *d* < *N*^*δ*^ where

$$\begin{array}{c} \delta <\frac{11}{9}-\frac{2}{9}\sqrt{4+18\gamma }. \end{array}$$

The construction of this paper is as follows. In Section 1, we intoduce the mechanisms and some results that will be used throughout this paper. In Section 2, we present the result on our attack theoretically. We also make a comparison with the previous attacks. Finally we conclude in Section 4.

## 2 Materials and methods

This section will discuss briefly on lattice basis reduction, Howgrave-Graham theorem, and useful lemmas that will be needed in this study.

### 2.1 Lattice

Suppose $\omega ,n\in Z^{+}$ with *ω* ≤ *n*. Let *v*_1_, ⋯, *v*_*ω*_ be linearly independent vectors in real numbers field. A lattice L is spanned by a set of linear combination {*v*_1_, ⋯, *v*_*ω*_} in the form

$$\begin{array}{c} L=\{\sum_{k=1}^{\omega }x_{k}u_{k}\;|\;x_{k}\in Z\} \end{array}$$

with the dimension of *ω*. The lattice is called full rank if the dimension *ω* = *n*. Thus, the determinant is calculated by taking the absolute value of the determinant of the matrix whose rows consist of {*v*_1_, ⋯, *v*_*ω*_} [16]. [17] formulated *LLL* algorithm to find a short basis vector in time polynomial.

**Theorem 1** [17] *Let* L *be the lattice generated by a set of basis* $\left\{v_{1},\ldots ,v_{\omega }\right\}$ *and has the dimension ω*. *The reduced basis* $\left\{b_{1},\ldots ,b_{\omega }\right\}$ *produced by the LLL algorithm satisfies*

$$\begin{array}{c} \left|\right|b_{1}\left|\right|\leq \left|\right|b_{2}\left|\right|\leq \cdots \leq \left|\right|b_{i}\left|\right|\leq 2^{\frac{\omega (\omega -1)}{4(\omega +1-i)}}\text{det}{\left(L\right)}^{\frac{1}{\omega +1-i}} \end{array}$$

*for all* 1 ≤ *i* ≤ *ω*.

Since its invention, *LLL* algorithm has been extensively applied in order to find reduced basis vectors in a lattice. For instance, [3] introduced a method to find a small roots of modular polynomial. Applying the *LLL* algorithm to find a reduced basis of the lattice generated by the modular polynomial, [3] managed to obtain the roots of the polynomial. Later, [18] described an alternative to Coppersmith’s method and he came out with the following theorem.

**Theorem 2** [18] *Let* $h\left(x_{1},\ldots ,x_{n}\right)\in Z\left[x_{1},\ldots ,x_{n}\right]$ *be a polynomial with at ω monomials*. *Suppose that* $h(x_{1}^{\left(0\right)},\ldots ,x_{n}^{\left(0\right)})\equiv 0\;(\text{mod}\;R)$ *where* $|x_{i}^{\left(0\right)}|<X_{i}$ for i=1,…,n, *and* $h(x_{1}X_{1},\ldots ,x_{n}X_{n})<\frac{R}{\sqrt{\omega }}$. *Then* $h(x_{1}^{\left(0\right)},\ldots ,x_{n}^{\left(0\right)})=0$ *holds over integers*.

Remark that our attack relies on a notable assumption that also had been used in some earlier proposed attacks such as [4, 15, 19].

**Assumption 1**. *The construction of LLL algorithm produces a number of coprime polynomials. The roots of these polynomials can be computed efficiently using the resultant technique*.

### 2.2 Approximation of primes in RSA

The following results by [20] show an approximation of the size of the primes and approximation of *N* − *ϕ*(*N*). These results will be used to approximate the bound for one of the variables in our polynomial.

**Lemma 1** *Let N* = *p*^2^*q with q* < *p* < 2*q*. *Then*

$$\begin{array}{c} 2^{-1/3}N^{1/3}<q<N^{1/3}<p<2^{1/3}N^{1/3}. \end{array}$$

**Lemma 2** *Let N* = *p*^2^*q with q* < *p* < 2*q*. *Then*

$$\begin{array}{c} 2N^{2/3}-N^{1/3}<N-\phi \left(N\right)<(2^{2/3}+2^{-1/3})N^{2/3}-2^{1/3}N^{1/3}. \end{array}$$

### 2.3 Prime sharing bits

The following lemma is reformulated from result [8]. It considers the case when the modulus *N* = *p*^2^*q* consists of two primes that share a known amount of their LSBs.

**Lemma 3** *Let N* = *p*^2^*q be the modulus and suppose that p* − *q* = 2^*b*^ *u for a known value of b*. *Let p* = 2^*b*^ *p*_1_ + *u*_0_ *and q* = 2^*b*^ *q*_1_ + *u*_0_ *where u*_0_ *is a solution to p*^3^ ≡ *N* (*mod* 2^*b*^). *If* $s_{0}\equiv u_{0}^{-1}\left(N-u_{0}^{3}\right)\;\left(mod\;2^{3b}\right)$ then *p*^2^ + *pq* − *p* = 2^3*b*^ *s* + *s*_0_ − *v where*

$$\begin{array}{c} v=(2^{b}p_{1}+2^{2b}p_{1}q_{1}-2^{b}p_{1}u_{0}-2u_{0}^{2}+u_{0}). \end{array}$$

*Proof*. Suppose that *p* − *q* = 2^*b*^ *u*. Then *q* = *p* − 2^*b*^ *u* and

$$\begin{array}{c} N=p^{2}q=p^{2}\left(p-2^{b}u\right)=p^{3}-2^{b}up^{2}. \end{array}$$ (1)

Hence, from (1), we obtain *p*^3^ ≡ *N* (mod 2^*b*^). Let *u*_0_ be a solution of the modular form *p*^3^ ≡ *N* (mod 2^*b*^). Then, *p* ≡ *u*_0_ (mod 2^*b*^) is a solution which implies that *p* = 2^*b*^ *p*_1_ + *u*_0_ where *p*_1_ is a positive integer. Now we have,

$$\begin{array}{c} q=p-2^{b}u=2^{b}p_{1}+u_{0}-2^{b}u=2^{b}\left(p_{1}-u\right)+u_{0}=2^{b}q_{1}+u_{0} \end{array}$$

where *q*_1_ = *p*_1_ − *u*. Using *N* = *p*^2^*q*, we get

$$\begin{array}{ccc} N & = & {\left(2^{b}p_{1}+u_{0}\right)}^{2}\left(2^{b}q_{1}+u_{0}\right)\\ & = & \left(2^{2b}p_{1}^{2}+2^{b+1}p_{1}u_{0}+u_{0}^{2})(2^{b}q_{1}+u_{0}\right)\\ & = & 2^{3b}p_{1}^{2}q_{1}+2^{2b}p_{1}^{2}u_{0}+2^{2b+1}p_{1}u_{0}q_{1}+2^{b+1}p_{1}u_{0}^{2}+2^{b}q_{1}u_{0}^{2}+u_{0}^{3}. \end{array}$$ (2)

From (2) we deduce

$$\begin{array}{ccc} 2^{2b}u_{0}\left(p_{1}^{2}+2p_{1}q_{1}\right)+2^{b}u_{0}^{2}\left(2p_{1}+q_{1}\right)+u_{0}^{3} & \equiv & N\;(\text{mod}\;2^{3b})\\ 2^{2b}u_{0}\left(p_{1}^{2}+2p_{1}q_{1}\right)+2^{b}u_{0}^{2}\left(2p_{1}+q_{1}\right) & \equiv & N-u_{0}^{3}\;\left(\text{mod}\;2^{3b}\right). \end{array}$$ (3)

Suppose gcd(*u*_0_, 2^3*b*^) = 1, we multiply (3) with $u_{0}^{-1}$ and get

$$\begin{array}{c} 2^{2b}\left(p_{1}^{2}+2p_{1}q_{1}\right)+2^{b}u_{0}\left(2p_{1}+q_{1}\right)\equiv u_{0}^{-1}\left(N-u_{0}^{3}\right)\;\left(\text{mod}\;2^{3b}\right) \end{array}$$

which can be rewritten as

$$\begin{array}{c} 2^{2b}\left(p_{1}^{2}+2p_{1}q_{1}\right)+2^{b}u_{0}\left(2p_{1}+q_{1}\right)=2^{3b}s+s_{0} \end{array}$$

where $s_{0}\equiv u_{0}^{-1}\left(N-u_{0}^{3}\right)\;\left(\text{mod}\;2^{3b}\right)$. Finally we have,

$$\begin{array}{ccc} p^{2}+pq-p & = & {\left(2^{b}p_{1}+u_{0}\right)}^{2}+\left(2^{b}p_{1}+u_{0}\right)\left(2^{b}q_{1}+u_{0}\right)-\left(2^{b}p_{1}+u_{0}\right)\\ & = & 2^{2b}p_{1}^{2}+2^{b+1}p_{1}u_{0}+u_{0}^{2}+2^{2b}p_{1}q_{1}+2^{b}p_{1}u_{0}+2^{b}q_{1}u_{0}+u_{0}^{2}-2^{b}p_{1}-u_{0}\\ & = & 2^{2b}\left(p_{1}^{2}+2p_{1}q_{1}\right)+2^{b}u_{0}\left(2p_{1}+q_{1}\right)-\left(2^{b}p_{1}+2^{2b}p_{1}q_{1}2^{b}p_{1}u_{0}-2u_{0}^{2}+u_{0}\right)\\ & = & 2^{3b}s+s_{0}-v \end{array}$$ (4)

where $v=(2^{b}p_{1}+2^{2b}p_{1}q_{1}-2^{b}p_{1}u_{0}-2u_{0}^{2}+u_{0}).$

## 3 The new attack

This section presents the attack on modulus *N* = *p*^2^*q* which works when there has a known amount of LSBs shared between the primes *p* and *q*.

**Theorem 3** *Let N* = *p*^2^*q be modulus such that p* − *q* = 2^*b*^ *u where* 2^*b*^ ≈ *N*^*α*^. *Let e be a public exponents satisfying e* ≈ *N*^*γ*^ *and ed* − *kϕ*(*N*) = 1. *Suppose that d* < *N*^*δ*^. *Then N can be factored in time polynomial if*

$$\begin{array}{c} \delta <\frac{11}{9}-\frac{2}{9}\sqrt{4+18\gamma }. \end{array}$$

*Proof*. Suppose we have public exponent *e* and key equation

$$\begin{array}{ccc} ed-k\phi (N) & = & 1\\ ed-k(p(p-1)(q-1)) & = & 1\\ ed-k(N-\left(p^{2}+pq-p\right)) & = & 1. \end{array}$$ (5)

Suppose that *p* − *q* = 2^*b*^ *u*. Then, from Lemma 3, *p*^2^ + *pq* − *p* can be rewritten in the form *p*^2^ + *pq* − *p* = 2^3*b*^ *s* + *s*_0_ − *v* where $s_{0}\equiv u_{0}^{-1}(N-u_{0}^{3})(\text{mod}\;2^{3b})$ and *u*_0_ is a solution of the modular equation *p*^3^ ≡ *N* (mod 2^*b*^). Thus, substitutes (4) from Lemma 3 into (5), we get

$$\begin{array}{c} ed-k(N-\left(2^{3b}s+s_{0}-v\right))=1. \end{array}$$

Rearranging the equation,

$$\begin{array}{c} ed-k\left(N-s_{0}\right)+k\left(2^{3b}s-v\right)-1=0. \end{array}$$ (6)

We transform (6) into

$$\begin{array}{c} a_{1}x_{1}+a_{2}x_{2}+x_{2}x_{3}+a_{3}=0. \end{array}$$

and we fix the coefficients and the variables of the polynomial as follows:

$$\begin{array}{c} \left\{ \begin{array}{cc} a_{1} & =e\\ a_{2} & =(N-s_{0}),\\ a_{3} & =-1 \end{array}\;\;\text{and}\;\;\{\begin{array}{cc} x_{1} & =d,\\ x_{2} & =k,\\ x_{3} & =2^{3b}s-v. \end{array}\right. \end{array}$$

Now, we consider the polynomial

$$\begin{array}{c} f\left(x_{1},x_{2},x_{3}\right)=a_{1}x_{1}+a_{2}x_{2}+x_{2}x_{3}+a_{3}. \end{array}$$

Then (*d*, *k*, 2^3*b*^ *s* − *v*) is a root of *f*(*x*_1_, *x*_2_, *x*_3_) and can be solved by using Coppersmith’s technique [3]. However, we choose to use the extended strategy of Jochemsz and May [21] due to its easier implementation. The following bounds will be needed:

- max(*e*_1_, *e*_2_) = *N*^*γ*^.
- max(*d*) < *X*_1_ = *N*^*δ*^.
- $k=\frac{e_{1}d_{1}-1}{\phi (N)}<X_{2}=N^{\gamma +\delta -1}$.
- *p* − *q* = 2^*b*^ *u* with 2^*b*^ ≈ *N*^*α*^ and $\alpha <\frac{2}{9}$.
- *p*^2^ + *pq* − *p* = 2^3*b*^ *s* + *s*_0_ − *v* with 2^3*b*^ *s* − *v* < *X*_3_ = *N*^2/3^.

The bounds of the variables are fixed as follows:

$$\begin{array}{c} X_{1}=N^{\delta },X_{2}=N^{\gamma +\delta -1},X_{3}=N^{2/3} \end{array}$$

Let $m,t\in Z^{+}$. The set *S* and *M* be defined as:

$$\begin{array}{c} S=\underset{0\leq j\leq t}{⋃}\left\{x_{1}^{i_{1}}x_{2}^{i_{2}}x_{3}^{i_{3}+j}|x_{1}^{i_{1}}x_{2}^{i_{2}}x_{3}^{i_{3}}\;\text{monomial}\;\text{of}\;f^{m-1}\right\} \end{array}$$

and the set

$$\begin{array}{c} M=\{\text{monomials}\;\text{of}\;x_{1}^{i_{1}}x_{2}^{i_{2}}x_{3}^{i_{3}}f\;\;|\;\;x_{1}^{i_{1}}x_{2}^{i_{2}}x_{3}^{i_{3}}\in S\}. \end{array}$$

Neglecting the coefficients, we find the expansion of polynomial *f*^*m*−1^(*x*_1_, *x*_2_, *x*_3_) satisfies

$$\begin{array}{c} f^{m-1}\left(x_{1},x_{2},x_{3}\right)=\sum_{i_{1}=0}^{m-1}\sum_{i_{2}=0}^{i_{1}}\sum_{i_{3}=0}^{m-1-i_{1}}x_{1}^{i_{1}}x_{2}^{i_{2}}x_{3}^{i_{3}} \end{array}$$ (7)

The monomials $x_{1}^{i_{1}}x_{2}^{i_{2}}x_{3}^{i_{3}}$ in (7) can be categorised as:

$$\begin{array}{c} x_{1}^{i_{1}}x_{2}^{i_{2}}x_{3}^{i_{3}}\in S\;\;\;\text{if}\;\;\;\{\begin{array}{c} i_{1}=0,\ldots ,m-1,\\ i_{2}=0,\ldots ,m-1-i_{1},\\ i_{3}=0,\ldots ,i_{2}+t. \end{array} \end{array}$$

Consequently, the monomials for set *M* are

$$\begin{array}{c} x_{1}^{i_{1}}x_{2}^{i_{2}}x_{3}^{i_{3}}\in M\;\;\;\text{if}\;\;\;\{\begin{array}{c} i_{1}=0,\ldots ,m,\;\;\;\\ i_{2}=0,\ldots ,m-i_{1},\\ i_{3}=0,\ldots ,i_{2}+t.\;\;\;\;\;\;\; \end{array} \end{array}$$

Define

$$\begin{array}{c} \begin{array}{cc} W & =||f(x_{1}X_{1},x_{2}X_{2},x_{3}X_{3}|{|}_{\infty }\\ & =\text{max}(|a_{1}|X_{1},|a_{2}|X_{2},X_{2}X_{3}). \end{array} \end{array}$$

Then *W* satisfies

$$\begin{array}{c} W\geq |a_{1}|X_{1}=ed\approx N^{\gamma +\delta }=N^{\gamma +\delta }. \end{array}$$ (8)

Next, define

$$\begin{array}{c} R=WX_{1}^{m-1}X_{2}^{m-1}X_{3}^{m-1+t}. \end{array}$$

Suppose that *a*_4_ is coprime with *R*. We want to work with a polynomial that has constant term 1, thus we define $f^{\prime }\left(x_{1},x_{2},x_{3}\right)=a_{4}^{-1}f\left(x_{1},x_{2},x_{3}\right)\;\text{mod}\;R$. Next, define the polynomials *g* and *h* as:

$$\begin{array}{c} \begin{array}{cc} g & =x_{1}^{i_{1}}x_{2}^{i_{2}}x_{3}^{i_{3}}f^{\prime }X_{1}^{m-1-i_{1}}X_{2}^{m-1-i_{2}}X_{3}^{m-1+t-i_{3}},\\ & \text{with}\;x_{1}^{i_{1}}x_{2}^{i_{2}}x_{3}^{i_{3}}\in S\\ h & =x_{1}^{i_{1}}x_{2}^{i_{2}}x_{3}^{i_{3}}R,\\ & \text{with}\;x_{1}^{i_{1}}x_{2}^{i_{2}}x_{3}^{i_{3}}\in M\backslash S. \end{array} \end{array}$$

The basis of a lattice L is built by using the coefficients of polynomials *g* and *h* with dimension

$$\begin{array}{c} \omega =\sum_{x_{1}^{i_{1}}x_{2}^{i_{2}}x_{3}^{i_{3}}\in M}1=\sum_{i_{1}=0}^{m}\sum_{i_{2}=0}^{m-i_{1}}\sum_{i_{3}=0}^{i_{2}+t}1=\frac{1}{6}\left(m+1\right)\left(m+2\right)\left(m+3t+3\right). \end{array}$$

In order to construct an upper triangular matrix, we perform the following ordering of the monomials: if $\sum i_{j}<\sum i_{j}^{\prime }$ then $x_{1}^{i_{1}}x_{2}^{i_{2}}x_{3}^{i_{3}}<x_{1}^{i_{1}^{\prime }}x_{2}^{i_{2}^{\prime }}x_{3}^{i_{3}^{\prime }}$ and the monomials are lexicographically ordered if $\sum i_{j}=\sum i_{j}^{\prime }$. The diagonal entries of the matrix are of the form

$$\begin{array}{c} \left\{ \begin{array}{cc} {\left(X_{1}X_{2}\right)}^{m-1}X_{3}^{m-1+t} & \text{for}\;\text{the}\;\text{polynomials}\;g\\ WX_{1}^{m-1+i_{1}}X_{2}^{m-1+i_{2}}X_{3}^{m-1+t+i_{3}} & \text{for}\;\text{the}\;\text{polynomials}\;h. \end{array}\right. \end{array}$$

Refer S1 Table in S1 Appendix for the coefficient matrix of *m* = 3 and *t* = 1.

Next, define

$$\begin{array}{c} s_{j}=\sum_{x_{1}^{i_{1}}x_{2}^{i_{2}}x_{3}^{i_{3}}\in M\backslash S}i_{j},\;\text{for}\;j=1,2,3. \end{array}$$ (9)

The determinant of L is then

$$\begin{array}{c} \text{det}(L)=W^{\left|M\backslash S\right|}X_{3}^{\left(m-1+t\right)|S|+\left(m-1+t\right)|M\backslash S|+s_{3}}\prod_{j=1}^{2}X_{j}^{\left(m-1\right)|S|+\left(m-1\right)|M\backslash S|+s_{j}} \end{array}$$

which can be simplified into

$$\begin{array}{c} \text{det}\left(L\right)=W^{\left|M\backslash S\right|}X_{3}^{\left(m-1+t\right)\omega +s_{3}}\prod_{j=1}^{2}X_{j}^{\left(m-1\right)\omega +s_{j}}. \end{array}$$

All the polynomials *g*(*x*_1_, *x*_2_, *x*_3_) and *h*(*x*_1_, *x*_2_, *x*_3_) and their combinations share the root (*d*, *k*, 2^3*b*^ *s* − *v*) modulo R. A new basis with short vectors is produced after applying the LLL algorithm to the lattice L. For *i* = 1, 2, let *f*_*i*_(*x*_1_ *X*_1_, *x*_2_ *X*_2_, *x*_3_ *X*_3_) be two short vectors of the reduced basis. Each *f*_*i*_ shares the roots (*d*, *k*, 2^3*b*^ *s* − *v*). Then by Theorem 3, we have

$$\begin{array}{c} \left|\right|f_{i}\left(x_{1}X_{1},x_{2}X_{2},x_{3}X_{3}\right)\left|\right|<2^{\frac{\omega (\omega -1)}{4(\omega -2)}}\text{det}{\left(L\right)}^{\frac{1}{\omega -2}}\;\;\text{for}\;i=1,2. \end{array}$$

In order to fulfill the condition of the bound proposed by [18], we force the polynomials *f*_*i*_ for *i* = 1, 2 to fulfill the condition of

$$\begin{array}{c} 2^{\frac{\omega (\omega -1)}{4(\omega -2)}}\text{det}{\left(L\right)}^{\frac{1}{\omega -2}}<\frac{R}{\sqrt{\omega }} \end{array}$$

which then can be transformed into det $\left(L\right)<R^{\omega }$, that is

$$\begin{array}{c} \begin{array}{cc} W^{\left|M\backslash S\right|}X_{3}^{\left(m-1+t\right)\omega +s_{3}}\prod_{j=1}^{2}X_{j}^{\left(m-1\right)\omega +s_{j}} & <{\left(WX_{1}^{m-1}X_{2}^{m-1}X_{3}^{m-1+t}\right)}^{\omega }\\ X_{3}^{s_{3}}\prod_{j=1}^{2}X_{j}^{s_{j}} & <W^{\omega -|M\backslash S|}. \end{array} \end{array}$$

Using *ω* = |*M*| and |*M*| − |*M*∖*S*| = |*S*|, we get

$$\begin{array}{c} \prod_{j=1}^{3}X_{j}^{s_{j}}<W^{\left|S\right|}. \end{array}$$ (10)

Using (9), we get

$$\begin{array}{c} \begin{array}{cc} s_{1} & =\sum_{i_{1}=0}^{m}\sum_{i_{2}=0}^{m-i_{1}}\sum_{i_{3}=0}^{i_{2}+t}i_{1}-\sum_{i_{1}=0}^{m-1}\sum_{i_{2}=0}^{m-1-i_{1}}\sum_{i_{3}=0}^{i_{2}+t}i_{1}\\ & =\frac{1}{6}m\left(m+1\right)\left(m+3t+2\right)\\ s_{2} & =\sum_{i_{1}=0}^{m}\sum_{i_{2}=0}^{m-i_{1}}\sum_{i_{3}=0}^{i_{2}+t}i_{2}-\sum_{i_{1}=0}^{m-1}\sum_{i_{2}=0}^{m-1-i_{1}}\sum_{i_{3}=0}^{i_{2}+t}i_{2}\\ & =\frac{1}{6}m\left(m+1\right)\left(2m+3t+4\right)\\ s_{3} & =\sum_{i_{1}=0}^{m}\sum_{i_{2}=0}^{m-i_{1}}\sum_{i_{3}=0}^{i_{2}+t}i_{3}-\sum_{i_{1}=0}^{m-1}\sum_{i_{2}=0}^{m-1-i_{1}}\sum_{i_{3}=0}^{i_{2}+t}i_{3}\\ & =\frac{1}{6}\left(m+1\right)\left(m^{2}+3mt+2m+3t^{2}+3t\right). \end{array} \end{array}$$

Correspondingly, we get

$$\begin{array}{c} \left|S\right|=\sum_{x_{1}^{i_{1}}x_{2}^{i_{2}}x_{3}^{i_{3}}\in S}1=\sum_{i_{1}=0}^{m-1}\sum_{i_{2}=0}^{m-1-i_{1}}\sum_{i_{3}=0}^{i_{2}+t}=\frac{1}{6}m\left(m+1\right)\left(m+3t+2\right). \end{array}$$

Set *t* = *τm*, then,

$$\begin{array}{c} \begin{array}{cc} s_{1} & =\frac{1}{6}\left(3\tau +1\right)m^{3}+o\left(m^{3}\right)\\ s_{2} & =\frac{1}{6}\left(3\tau +2\right)m^{3}+o\left(m^{3}\right)\\ s_{3} & =\frac{1}{6}\left(3\tau^{2}+3\tau +1\right)m^{3}+o\left(m^{3}\right)\\ \left|S\right| & =\frac{1}{6}\left(3\tau +1\right)m^{3}+o\left(m^{3}\right) \end{array} \end{array}$$

Using this, and after simplifying by *m*^3^, the inequality (10) transform into

$$\begin{array}{c} X_{1}^{\frac{1}{6}\left(3\tau +1\right)}X_{2}^{\frac{1}{6}\left(3\tau +2\right)}X_{3}^{\frac{1}{6}\left(3\tau^{2}+3\tau +1\right)}<W^{\frac{1}{6}\left(3\tau +1\right)} \end{array}$$

Substituting the values of *X*_1_, *X*_2_, *X*_3_ and *W* from (8) we get

$$\begin{array}{c} \left(\delta \right)\left(\frac{1}{6}\left(3\tau +1\right)\right)\left(\gamma +\delta -1\right)\left(\frac{1}{6}\left(3\tau +2\right)\right)\left(\frac{2}{3}\right)\left(\frac{1}{6}\left(3\tau^{2}+3\tau +1\right)\right)<\left(\gamma +\delta \right)\left(\frac{1}{6}\left(3\tau +1\right)\right) \end{array}$$

or equivalently,

$$\begin{array}{c} \frac{1}{3}\tau^{2}+\frac{1}{6}(3\delta -1)\tau +\frac{1}{36}\left(6\gamma +12\delta -8\right)<0. \end{array}$$

Differentiate the equation above with respect to *τ*, we get the optimal value $\tau =\frac{-3\delta +1}{4}$, this reduces to

$$\begin{array}{c} -27\delta^{2}+66\delta +24\gamma -35<0 \end{array}$$

which is valid if

$$\begin{array}{c} \delta <\frac{11}{9}-\frac{2}{9}\sqrt{4+18\gamma }. \end{array}$$

Under this condition of *δ*, we find our reduced polynomials *f*, *f*_1_, *f*_2_ with the root of (*d*, *k*, 2^3*b*^ *s* − *v*). By Assumption 1 in Section 2, the solution of the roots can be extracted using resultant technique. By using the third root 2^3*b*^ *s* − *v*, we compute *p*^2^ + *pq* − *p* = 2^3*b*^ *s* + *s*_0_ − *v*. This value is then used to find *ϕ*(*N*) and since *ϕ*(*N*) = *p*(*p* − 1)(*q* − 1) we can factor out *p* by taking the gcd(*N*, *ϕ*(*N*)). By knowing the value of *p*, we can factor the modulus *N*.

### 3.1 Comparison with the former attack

We compare our bounds with these three former attacks, [14, 15 and 19] that also work on modulus *N* = *p*^*r*^ *q* but we specifically consider the case when *r* = 2. Their attacks focused on the RSA key equation *ed* − *kϕ*(*N*) = 1 where *ϕ*(*N*) = *p*^*r*−1^(*p*^*r*^ − 1)(*q* − 1). Note that in these former attacks, their primes do not share any amount of LSBs. Their bounds depend only on the value of *r*. We compare the results with various values of *γ* = log_*N*_(*e*). Our corollary is as follow.

**Corollary 1** *Let N* = *p*^2^*q be the modulus where q* < *p* < 2*q*. *Let e be a public exponent satisfying ed* − *kϕ*(*N*) = 1 *for ϕ*(*N*) = *p*(*p* − 1)(*q* − 1). *Suppose that d* < *N*^*δ*^. *Then N can be factored in time polynomial if*

$$\begin{array}{c} \delta <\frac{11}{9}-\frac{2}{9}\sqrt{4+18\gamma }. \end{array}$$

Note that the bounds for *δ* of [14, 15 and 19] remain fixed because their bounds only depend on the value of *r* = 2. We describe their bound for *d* as in the Table 1 below.

**Table 1:** Bounds for *d* from the former attacks.

Former attack	Bound
[14]	$d<N^{\text{max}\{\frac{r}{{\left(r+1\right)}^{2}},\frac{{\left(r-1\right)}^{2}}{{\left(r+1\right)}^{2}}\}}=N^{0.22}$
[15]	*d* < *N*^0.39^
[19]	$d<N^{\frac{r(r-1)}{{\left(r+1\right)}^{2}}}=N^{0.22}$

Table 2 shows that our bound improves the previous bounds. The value of *δ* increases inversely proportional to the value of *γ*.

**Table 2:** Comparison of the new result with methods from [14, 15, 19].

*γ* = log_*N*_(*e*)	*γ* = 0.6	*γ* = 0.5	*γ* = 0.4	*γ* = 0.3
Bound of *δ*				
[14]	0.22	0.22	0.22	0.22
[15]	0.39	0.39	0.39	0.3
[19]	0.22	0.22	0.22	0.22
Our bound in Corollary 1	0.36	0.42	0.47	0.54

**Remark 1** In Corollary 1, it states that *N* can be factored if

$$\begin{array}{c} \delta <\frac{11}{9}-\frac{2}{9}\sqrt{4+18\gamma }. \end{array}$$

Suppose *e* = *N*^*γ*^. We have

$$\begin{array}{c} ed=1+k\phi (N)>\phi (N)\approx N. \end{array}$$

then

$$\begin{array}{c} d>\frac{N}{e}=N^{1-\gamma }. \end{array}$$

From the fact that

$$\begin{array}{c} \delta >1-\gamma \end{array}$$

and combining it with results from Corollary 1, $\delta <\frac{2}{3}-\frac{1}{2}\gamma$, we have

$$\begin{array}{cc} 1-\gamma & <\frac{11}{9}-\frac{2}{9}\sqrt{4+18\gamma }. \end{array}$$

From here, we can find the bound for the positive value of *γ*. A direct calculation shows that $\gamma <\frac{2}{3}$. For small values of *γ*, this translates into *d* ≈ *N*.

## 4 Conclusion

We describe an attack related to partial key exposure. Our attack works upon the modulus *N* = *p*^2^*q* where the primes share an amount of LSB(s). Based on the result of Nitaj et al. [8], we reformulate their lemma within our theorem and find the substitution for *p*^2^ + *pq* − *p* which is the value of *N* − *ϕ*(*N*). We use the result from our lemma in our theorem which then yields a set of integer polynomials. By applying the extended strategy of Jochemsz and May, one is able to determine the small roots of our integer polynomial and thus factoring the modulus *N*. We show that *N* can be factored when *d* < *N*^*δ*^ for $\delta <\frac{2}{3}+\frac{3}{2}\alpha -\frac{1}{2}\gamma$ where $0<\gamma <\frac{2}{3}$. As such, we manage to improve the bounds of some previous attacks on the modulus *N* = *p*^2^*q*.

## Supporting information

S1 Appendix (PDF)
