# Supplementary material for: Analytical cryptanalysis upon N = p2q utilizing Jochemsz-May strategy
Source: PLoS One. 2021 Mar 24;16(3):e0248888. doi: 10.1371/journal.pone.0248888 (PMC7990206; doi:10.1371/journal.pone.0248888)
Supplement: S1 Appendix — (PDF) [file pone.0248888.s001.pdf]

S1 Appendix

$M =$

	1	x_3	x_2	x_2x_3	$x_2x_3^2$	x_2^2	$x_2^2x_3$	$x_2^2x_3^2$	$x_2^2x_3^3$	x_1	x_1x_3	x_1x_2	$x_1x_2x_3$	$x_1x_2x_3^2$	x_1^2	$x_1^2x_3$
$g_{0,0,0}$	A	**	**							**						
$g_{0,0,1}$		A		**	**						**					
$g_{0,1,0}$			A			**	**					**				
$g_{0,1,1}$				A			**	**					**			
$g_{0,1,2}$					A			**	**					**		
$g_{0,2,0}$						B										
$g_{0,2,1}$							B									
$g_{0,2,2}$								B								
$g_{0,2,3}$									B							
$g_{1,0,0}$										A		**	**	**		
$g_{1,0,1}$											A		**	**	**	
$g_{1,1,0}$												B				
$g_{1,1,1}$													B			
$g_{1,1,2}$														B		
$g_{2,0,0}$															B	
$g_{2,0,1}$																B

S1 Table. The coefficient matrix for the case $m = 2$ and $t = 0$.

Remark that the values of A and B are as follows:

$$A = X_1X_2X_3^2$$
$$B = X_1^{i_1}X_2^{i_2}X_3^{i_3}R.$$
